# Supplementary material for: The Impact of GLP-1 RAs and DPP-4is on Hospitalisation and Mortality in the COVID-19 Era: A Two-Year Observational Study
Source: Biomedicines. 2023 Aug 18;11(8):2292. doi: 10.3390/biomedicines11082292 (PMC10452157; doi:10.3390/biomedicines11082292)
Supplement: Supplementary file 1 [file biomedicines-11-02292-s001.zip › biomedicines-2552584-supplementary.pdf]

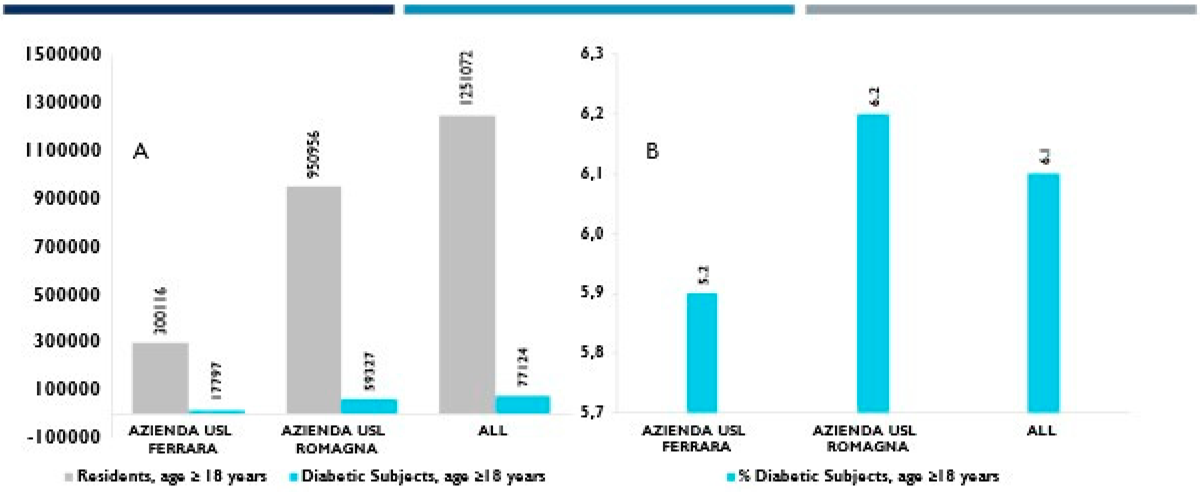

Figure S1. Prevalence of diabetes in the overall adult subjects in the districts of Ferrara and Romagna (Panel A), percentage of diabetic subjects in the districts of Ferrara and Romagna and overall average of diabetic subjects (Panel B)

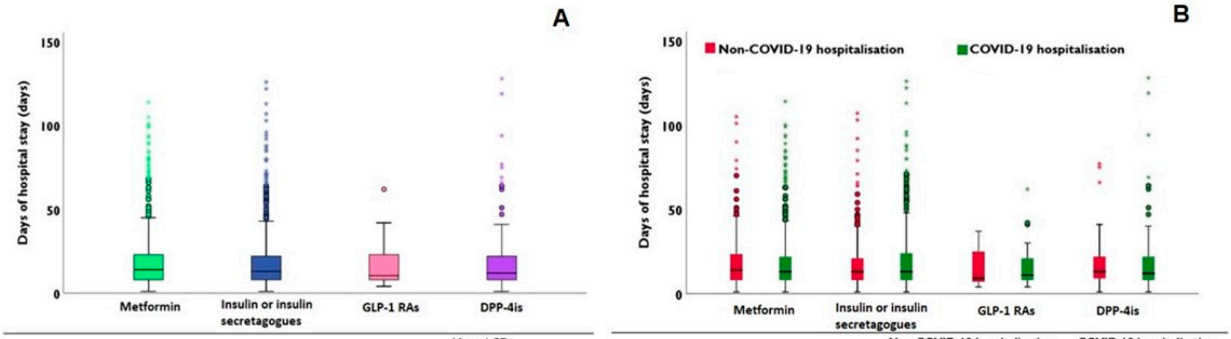

	Mean ± SD
Metformin	18.9 ± 17.7
Insulin or Insulin secretagogues	18.6 ± 17.9
GLP-1 RAs	15.9 ± 12.0
DPP-4is	17.3 ± 17.0

	Non-COVID-19 hospitalisation	COVID-19 hospitalisation
Metformin, Mean ± SD	18.7 ± 16.3	19.0 ± 18.2
Insulin or Insulin secretagogues, Mean ± SD	17.4 ± 16.1	19.4 ± 18.9
GLP-1 RAs, Mean ± SD	14.7 ± 10.8	16.6 ± 12.8
DPP-4is, Mean ± SD	17.1 ± 13.8	17.5 ± 18.6

Figure S2. Evaluation of the length of stay in terms of days in the subgroup of patients treated with the four main antidiabetic treatments (Panel A); length of stay among the same four subgroups of inpatients and comparison between COVID-19 and non-COVID-19 subjects (Panel B)

Table S1. The institutions serving the districts of Ferrara and Romagna participating to the study

Institution	District	City (Province)
S. Anna Hospital	Ferrara	Cona (Ferrara)
SS.ma Annunziata Hospital	Ferrara	Cento (Ferrara)
Mazzolani-Vandini Hospital	Ferrara	Argenta (Ferrara)
Ospedale del Delta	Ferrara	Lagosanto (Ferrara)
Maurizio Bufalini Hospital	Romagna	Cesena (Forlì-Cesena)
Morgagni-Pierantoni Hospital	Romagna	Forlì (Forlì-Cesena)
S. Maria delle Croci	Romagna	Ravenna (Ravenna)
Ospedale degli Infermi	Romagna	Faenza (Ravenna)
Umberto I Hospital	Romagna	Lugo (Ravenna)
Ospedale Infermi	Romagna	Rimini (Rimini)
Ceccarini Hospital	Romagna	Riccione (Rimini)

Table S2. MACEs and antidiabetic drugs

Antidiabetic treatment	Total	MI	CVA	HF	MD
Metformin. n (%)	30238	131 (0.4)	429 (1.4)	93 (0.3)	10 (0.0)
Insulin or Insulin secretagogues. n (%)	14739	152 (1.0)	427 (2.9)	104 (0.7)	20 (0.1)
GLP-1 RAs. n (%)	127	3 (2.4)	1 (0.8)	0 (0.0)	0 (0.0)
GLP-1 RAs + Metformin. n (%)	910	6 (0.7)	10 (1.1)	2 (0.2)	0 (0.0)
GLP-1 RAs + Insulin or Insulin secretagogues. n (%)	190	1 (0.5)	2 (1.1)	2 (1.1)	1 (0.5)
DPP-4is. n (%)	1169	17 (1.5)	45 (3.8)	10 (0.9)	5 (0.4)
DPP-4is + Metformin. n (%)	2037	10 (0.5)	24 (1.2)	7 (0.3)	0 (0.0)
DPP-4is + Insulin or Insulin secretagogues. n (%)	1095	24 (2.2)	42 (3.8)	12 (1.1)	0 (0.0)
Other drug combinations or other drugs. n (%)	26259	229 (0.9)	490 (1.9)	93 (0.4)	14 (0.1)
	**Total**	**2-point MACE**	**3-point MACE**	**4-point MACE**	
Metformin. n (%)	30238	551 (1.8)	828 (2.7)	893 (3.0)	
Insulin or Insulin secretagogues. n (%)	14739	571 (3.9)	1214 (8.2)	1268 (8.6)	
GLP-1 RAs. n (%)	1227	23 (1.9)	37 (3.0)	39 (3.2)	
DPP-4is. n (%)	4301	160 (3.7)	358 (8.3)	370 (8.6)	
Other drug combinations or other drugs. n (%)	26259	702 (2.7)	1169 (4.5)	1224 (4.7)	

Prevalence of singular MACEs in each subgroup of diabetic subjects according to their antidiabetic treatment (upper panel). Prevalence of 2-point MACE. 3-point MACE and 4-point MACE in the subgroups of patients treated with the 5 main antidiabetic drugs/combinations of drugs (lower panel); 2-point MACE (non-fatal MI, non-fatal CVA); 3-point MACE (non-fatal MI, non-fatal CVA, and HF with or without CS); 4-point MACE (non-fatal MI, non-fatal CVA, HF with or without CS, and MD).
